# Annexin A7 suppresses lymph node metastasis of hepatocarcinoma cells in a mouse model

**DOI:** 10.1186/1471-2407-13-522

**Published:** 2013-11-04

**Authors:** Yanling Jin, Shaoqing Wang, Wenjing Chen, Jun Zhang, Bo Wang, Hongwei Guan, Jianwu Tang

**Affiliations:** 1 Department of Pathology, First Affiliated Hospital of Dalian Medical University, Dalian 116011, Liaoning Province, China; 2 Department of Pathology, Dalian Medical University, 9 West Lvshun Southern Road, Dalian 116044, P.R. China

**Keywords:** Annexin A7, Lymph node metastasis, HCC, Gene transfection, Animal experiment

## Abstract

**Background:**

Hepatocellular carcinoma (HCC) is one of the leading causes of cancer death in China. This study investigated the effects of Annexin A7 (ANXA7) on the inhibition of HCC lymph node metastasis in a mouse model.

**Methods:**

The stable knockup and knockdown of Annexin A7-expressing HCC cells using Annexin A7 cDNA and shRNA vectors, respectively, were injected into a mouse footpad to establish primary and metastatic tumors in mice. On the 14th, 21st, and 28th days after HCC cells inoculation, the mice were sacrificed for inspection of primary and secondary tumors and immunohistochemistry of Annexin A7 expression.

**Results:**

The lymph node metastasis rate of the F_ANXA7-control_ group was 77%, and the lymph node metastasis rate of the F_ANXA7-down_ group was 100% (*p* < 0.05). In contrast, the lymph node metastasis rate of the P_ANXA7-up_ group was 0% and that of the P_ANXA7-control_ group was 36% (*p* < 0.05). Furthermore, immunohistochemistry experiments revealed that the subcellular localization of Annexin A7 protein in both primary and lymph node-metastasized tumors was mainly in the cytosol. In addition, the expression of the 47 kDa and 51 kDa isoforms of Annexin A7 protein changed during tumor progression.

**Conclusion:**

This study indicated that Annexin A7 expression was able to inhibit HCC lymph node metastasis, whereas knockdown of Annexin A7 expression significantly induced HCC metastasis to local lymph nodes.

## Background

Hepatocellular carcinoma (HCC), the most common type of liver cancer, is a significant health problem in the world due to its high incidence and mortality rate. HCC accounts for more than 700,000 new cases and over 500,000 deaths each year worldwide. HCC is heterogeneous and a highly aggressive malignancy; to date, there are no effective means for a cure, due to high invasion, early metastasis, and high tumor recurrence after surgery or interventional treatment. Therefore, early detection and prevention of HCC and the control of HCC metastasis are urgently needed to improve HCC prognosis. The risk factors for HCC include heavy alcohol consumption, hepatitis B and C, aflatoxin, liver cirrhosis, hemochromatosis, and type 2 diabetes; thus, eradication of these risk factors could significantly reduce HCC risk. Furthermore, HCC progression, like metastasis, contributes to most human cancer deaths. Mechanistically, metastasis involves multiple processes, such as tumor cell proliferation, invasion, transportation, arrest, adherence, extravasation, settling-down, and growth in secondary sites [1]. Lymph node metastasis of a tumor is considered as an important factor that is involved in tumor progression.

However, the underlying molecular mechanisms involved in lymph node metastasis of tumors remain undefined. To date, a number of genes have been identified that modulate lymphatic tumor metastasis when they are highly expressed in certain tumor cells, such as Ezrin [2], AF1QN [3], MMP-11 [4], or Annexin A7 [5,6]. Annexin A7 is a member of the multifunctional calcium/phospholipid-binding annexin family that functions as a Ca^2+^-activated GTPase with membrane fusion properties. A spliced cassette exon generally induces two isoforms of Annexin A7 (47 kDa and 51 kDa). The 47 kDa isoform is present in all tissues except for skeletal muscle, while the 51 kDa isoform is exclusively present in the brain, heart, and skeletal muscle. Protein structural analysis indicates that Annexin A7 is a membrane binding protein with diverse properties, such as voltage-sensitive calcium channel activity, ion selectivity, and membrane fusion properties. However, the precise molecular action of this protein is unclear, especially in HCC cell metastasis.

Our previous study demonstrated that Annexin A7 mRNA expression is 3.48-fold greater in Hca-F cells than in Hca-P cells after cDNA microarray and gene chip assays [5]; in addition, Annexin A7 protein expression is three times higher in Hca-F cells than in Hca-P cells, as shown by using two-dimensional differential in-gel electrophoresis (2-D DIGE) minimal labeling analysis [6], indicating that at the mRNA and protein levels, Annexin A7 was more highly expressed in the Hca-F cell line with a high potential for lymphatic metastasis than in the Hca-P cell line with low potential for lymphatic metastasis. These data suggest that Annexin A7 may be a lymph node metastasis-associated gene and may play a key role in HCC involved with lymph node metastasis. Therefore, to gain more insight into the potential mechanisms and associated genes that are involved in HCC lymph node metastasis, we proposed the current study by using two mouse hepatocarcinoma ascites syngeneic cell lines, Hca-F (lymph node metastasis rate >70%) and Hca-P (lymph node metastasis rate <30%) [7-9] to assess the effects of Annexin A7 on the regulation of HCC cell metastasis in an inbred Chinese 615 mouse model of metastasis.

## Methods

### Ethics statement

Inbred Chinese 615 mice (6–8 weeks old, SCXK (LIAO) 2008–0002) provided by the Experimental Animal Center of Dalian Medical University. Mice were maintained under standard conditions and treated according to the institutional guidelines for the use of laboratory animals. All animal experiments were conducted in accordance with protocols approved by the Experimental Animal Ethical Committee of Dalian Medical University (Permit Number: L2012012).

### Cell lines and culture

Both Hca-F and Hca-P cells were established and maintained in our laboratory [5,6]. Cells were grown *in vitro* and then inoculated at 2 × 10^6^ cells in a 0.2 ml cell suspension into each inbred Chinese 615 mouse and grown in the mouse abdominal cavity for 7 days. These cells were drawn and injected again into another inbred Chinese 615 mouse and grown for 5 days. Then, those cells were routinely cultured in RPMI-1640 (Gibco, CA, USA) supplemented with 10% fetal bovine serum (PAA, CA, USA) at 37°C in a humidified incubator with 5% CO_2_ and 95% air. For Annexin A7 gene transfection subcell populations, they were maintained in the same conditions except for cultivating in RPMI-1640 supplemented with 10% FBS and 400 μg/ml G418 (Calbiochem, CA, USA).

### Expression vector construction and gene transfection

To knockdown Annexin A7 expression in Hca-F cells, we constructed three shRNA plasmids (Annexin A25, Annexin A59, and Annexin A507) using mouse Annexin A7 cDNA (accession # NM_009674.3). The target sequences for Annexin A25 were 5′-GATCCGTCAGAATTGAGTGGGAATTTCAAGAGAATTCCCACTCAATTCTGACTTTTTTGGAAA-3′ and 5′-AGCTTTTCCAAAAAAGTCAGAATTGAGTGGGAATTCTCTTGAAATTCCCACTCAATTCTGACG-3′. The target sequences for Annexin A59 were 5′-GATCCGCGACTCTACTATTCCATGATTCAAGAGATCATGGAATAGTAGAGTCGTTTTTTGGAAA-3′ and 5′-AGCTTTTCCAAAAAACGACTCTACTATTCCATGATCTCTTGAATCATGGAATAGTAGAGTCGCG-3′. The target sequences for Annexin A507 were 5′-GATCCGCAAAGCAATGAAAGGGTTCTCAAGAGAAACCCTTTCATTGCTTTGCGGTTTTTTGGAAA-3′ and 5′-AGCTTTTCCAAAAAACCGCAAAGCAATGAAAGGGTTTCTCTTGAGAACCCTTTCATTGCTTTGCG-3′. The primer for M13F was 5′-GTTTTCCCAGTCACGAC-3′. After annealing into double-stranded DNA, these shRNA oligonucleotides were then inserted into pSilencer™ 3.1-H1 neo plasmids (Shanghai ZJ Bio-Tech, China) using the BamHI and HindIII sites and transfected into *Escherichia coli* DH5α for amplification. After sequence confirmation, these plasmids were then used for stable gene transfection. To increase Annexin A7 expression in Hca-P cells, the Annexin A7 gene was amplified, and BamH 1 and EcoR I enzymes were used. The pcDNA3.1-Annexin A7 expressing vectors was constructed.

Hca-F and Hca-P cells with a density of 1 × 10^6^ cells/well were cultured in six-well plates with serum-free RPMI-1640 medium. These plasmids and “empty” vector were transfected into Hca-F cells using 15 μL of Lipofectamine 2000 (Invitrogen, CA, USA) according to the manufacturer’s instructions and cultivated in RPMI-1640 medium. After that, the cells were cultured in RPMI-1640 medium containing G418 (400 μg/mL) to establish a stable subcell population. After 16 days, approximately 85–95% of the cells were eliminated, and the remaining cells were continuously cultured in RPMI-1640 medium containing G418 (400 μg/mL) for our experiments. Hca-F and Hca-P cells were each divided into three groups:

♦ Unmanipulated Hca-F cells were used as the control (Hca-F).

♦ Empty plasmids were transfected into Hca-F cells (F_ANXA7-control_).

♦ shRNA-ANXA7 plasmids were transfected into Hca-F cells (F_ANXA7-down_).

♦ Unmanipulated Hca-P cells were used as the control (Hca-P).

♦ Empty plasmids were transfected into Hca-P cells (P_ANXA7-control_).

♦ pcDNA3.1-ANXA7 plasmids were transfected into Hca-P cells (P_ANXA7-up_).

### RNA isolation and reverse transcription polymerase chain reaction (RT-PCR)

Total RNA was isolated from the cultured cells using Trizol (Invitrogen, USA), according to the manufacturer’s instructions. After RNA isolation, cDNA was prepared from each sample by using 2 μg of total RNA for reverse transcription in a mixture, consisting of 4 μL of 5× RT-buffer, 2.5 μM oligo(dT) primers, 5 mM dNTPs, 20 U RNasin, and reverse transcriptase (Promega, CA, USA). The PCR mixture contained 4 μL of 10× buffer, 0.8 μL of 10 mM dNTP, 3.2 μL of 25 mM MgCl_2_, 0.5 μL of each primer, and 0.3 μL of 5 U Taq DNA gold Polymerase (Takara, CA, Japan). The PCR conditions were as follows: 95°C for 5 min; 30 cycles of 94°C for 15 s, 57°C for 20 s, and 72°C 60 s; and a final extension at 72°C for 10 min. The Annexin A7 primers were 5′-TGTCTAACCGTTCCAATGACC-3′ (upstream) and 5′-GGATTCATCCGTTCCCAGTC-3′ (downstream).

### Protein extraction and western blot

Total cellular protein was extracted using a buffer containing lysate, DTT, PMSF, and cocktail (Roche, CA, China). Next, protein samples were fractionated by using 12% SDS-PAGE. Annexin A7 antibody (A4475, Sigma, USA) was used at a dilution of 1:1500 for western blot, and GAPDH antibody (Kangchen, CA, China) was used at a dilution of 1:7500. The secondary antibody (Zhongshan Golden Bridge, CA, China) was used at a dilution of 1:4000 for both Annexin A7 and GAPDH. Positive protein bands were visualized by using electrochemiluminescence reagents (Santa Cruz Biotechnologies, CA, USA) and quantified by using densitometry (Bio-Rad, CA, USA).

### Animal experiments

A total of 84 Inbred Chinese 615 mice were provided by the experimental animal housing facility at Dalian Medical University. The mouse model of HCC cell lymphatic metastasis was established by injection of HCC cells into the left footpad of inbred Chinese 615 mice (called “primary tumor”). The number of cells injected into each mouse was 2 × 10^6^/0.05 ml. On the 14th, 21st, and 28th days after tumor cell inoculation, the mice were sacrificed and examined. During these periods of time, all of the inoculated tumor cells developed xenografts in the footpad for Hca-F, F_ANXA7-control_, F_ANXA7-down_, Hca-P, P_ANXA7-control_, and P_ANXA7-up_ groups. Some of the mice also developed metastatic tumors in different regional lymph nodes, which were called “metastasized lymph nodes”, including popliteal, inguinal, and iliac artery lymph nodes. Lymph nodes volumes (V) were calculated by the formula V = L × W^2^ × 0.5. All primary and secondary tumor xenografts were collected for processing by hematoxylin and eosin (HE) staining. In addition, protein samples from the primary tumor were extracted for western blot analysis of Annexin A7 protein expression. Meanwhile, protein samples from the primary tumor in the Hca-F cell group on the 14th, 21st, and 28th days were called “F_14_” group, “F_21_” group, and “F_28_” group, respectively; protein samples from the primary tumor in the F_ANXA7-down_ cell group on the 28th day were called the “F_ANXA7-down28_” group; protein samples from the primary tumor in the Hca-P cell group on the 14th, 21st, and 28th days were called “P_14_” group, “P_21_” group, and “P_28_” group, respectively; protein samples from the primary tumor in the P_ANXA7-up_ cell group on the 28th day were called the “P_ANXA7-up28_” group. The popliteal, iliac artery, and inguinal lymph nodes were collected for HE staining and immunohistochemistry to examine potential secondary tumors and Annexin A7 expression under a microscope.

### Immunohistochemistry

Immunohistochemistry was used to detect Annexin A7 expression in the mouse xenografts. A mouse monoclonal antibody against Annexin A7 was used at a dilution of 1:200. A rabbit anti-mouse secondary antibody (Santa Cruz Biotechnology, CA, USA) was used at a 1:1000 dilution. Annexin A7 protein expression was quantified based on staining intensity and uniformity of nuclear/cytoplasmic staining. The percentage of staining was scored as 1, ≤10%; 2, 11-50%; 3, 51-75%; and 4, >75%. Staining intensity was scored as 0, no staining; 1, stramineous color; 2, yellow color; and 3, brown. Next, these scores were combined to give a final score for each section as - to +++ (-, no signal; +, weak indeterminate signal; ++, moderate signal; +++, strong signal). All sections were stained simultaneously, together with the appropriate positive and negative controls.

### Statistical analysis

Statistical analysis was performed using the χ^2^-test and standard one-way analysis of variance (ANOVA) or one-way ANOVA for repeated measures. Immunohistochemical data were compared using a rank-sum test. A *p*-value < 0.05 was considered statistically significant.

## Results

### Establishment of stable knockup and knockdown of Annexin A7-expressing HCC cells

**Figure 1:**
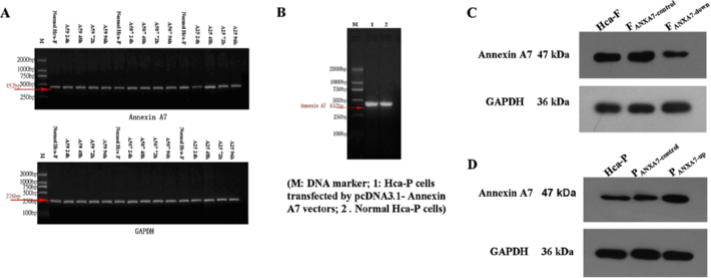
**Expression of Annexin A7 mRNA and protein. (A)** RT-PCR analysis of Annexin A7 gene silencing in Hca-F cells using three shRNA constructs (Annexin A25, Annexin A59, and Annexin A507). pSilencer-Annexin A25 shRNA was more potent than pSilencer-Annexin A507 and pSilencer-Annexin A59 shRNA. **(B)** RT-PCR analysis of Annexin A7 mRNA expression in Hca-P cells. **(C)** Western blot analysis of Annexin A7 expression in Hca-F, F_ANXA7-control_, and F_ANXA7-down_ cells_._**(D)** Western blot analysis of Annexin A7 expression in Hca-P, P_ANXA7-control_, and P_ANXA7-up_ cells.

In this study, we established stable cells that expressed knockdown of Annexin A7 in Hca-F cell lines. The RT-PCR results showed that different constructs of shRNA had different knockdown efficiencies of Annexin A7 expression. While, the transfection efficiency of pSilencer-Annexin A25 shRNA was better than those of Annexin A59 and Annexin A507 in silencing Annexin A7 gene expression in Hca-F cells 24 h after transfection (Figure 1A). The Δct values of Hca-F, F_ANXA7-control_, and F_ANXA7-down_ cells were 2.02, 3.06, and 4.88, respectively. Western blot results indicated that the expression of Annexin A7 in F_ANXA7-down_ was significantly lower than F_ANXA7-control_, but Annexin A7 expression in Hca-F showed no difference compared to F_ANXA7-control_ (Figure 1C)_._ These data showed that Hca-F cells had downregulated expression of Annexin A7, not only at the gene level but also at the protein level.

The RT-PCR data showed that the Δct value of Annexin A7 gene expression in Hca-P cells was 0.54, and in P_ANXA7-up_ cells it was 0.24 (Figure 1B). Western blot analysis showed that Annexin A7 expression in P_ANXA7-up_ cells was greater than in P_ANXA7-control_, but Annexin A7 expression in Hca-P showed no difference compared to P_ANXA7-control_ (Figure 1D). These data illustrated that the pcDNA3.1-Annexin A7 expression vector was successfully constructed and stably expressed in Hca-P cells, indicating that Hca-P cells had upregulated expression of Annexin A7 both at the gene and protein levels after gene transfection.

### Effects of Annexin A7 on the regulation of HCC cell lymph node metastasis in a mouse model

**Figure 2:**
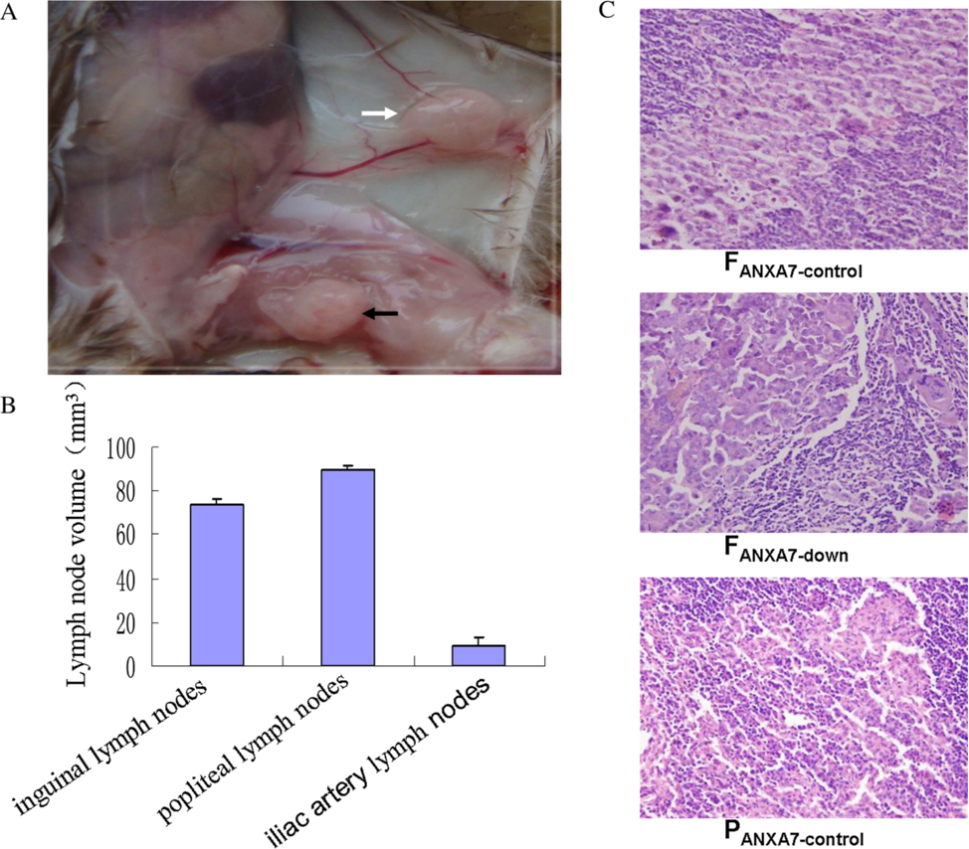
**Metastatic lymph nodes. (A)** The white arrow indicates the metastatic inguinal lymph node, and the black arrow indicates a metastatic popliteal lymph node. **(B)** The volumes of metastatic popliteal, inguinal, and iliac artery lymph nodes. **(C)** The metastatic lymph nodes of F_ANXA7-control_, F_ANXA7-down_, and P_ANXA7-control_ were fixed in 10% neutral-buffered formalin, paraffin-embedded, and cut into 4-μm sections for HE staining (400×).

The above results evidently indicate that Annexin A7 was successfully downregulated in Hca-F cells and upregulated in Hca-P cells. After the two stable cell lines were established, they were injected into the footpads of inbred Chinese 615 mice to ensure that all the mice developed a “primary tumor”. The metastatic tumor xenografts developed in the lymph nodes (Figure 2A). The results showed that the lymph node metastasis rate of the F_ANXA7-control_ group was 77%, and that of the F_ANXA7-down_ group was 100% (*p* < 0.05, Table 1), indicating that after downregulation of the Annexin A7 gene in Hca-F cells with high lymphatic metastasis potential, the lymph node metastasis rate was increased significantly *in vivo*. In contrast, the lymph node metastasis rate of the P_ANXA7-up_ group was 0% and that of the P_ANXA7-control_ group was 36% (*p* < 0.05, Table 1), demonstrating that Annexin A7 protein expression significantly reduced lymph node metastasis upon upregulation of the Annexin A7 gene in Hca-P cells.

**Table 1:** Lymph node metastasis rates in hepatocarcinoma after altered Annexin A7 expression

	**Mice with inoculated tumors (n)**	**Mice with lymph node metastases (n)**	**Lymph node metastases rate (%)**	***P*-value**
F_ANXA7-control_	13	10	77	0.018
F_ANXA7-down_	34	34	100	
P_ANXA7-control_	11	4	36	0.005
P_ANXA7-up_	26	0	0	

The metastasized lymph nodes included the popliteal, inguinal, and iliac artery lymph nodes. The data indicated that the volumes of the popliteal and inguinal lymph nodes were significantly larger than the iliac artery lymph nodes (*p* < 0.05), and the volumes of the popliteal lymph nodes were significantly larger than the inguinal lymph nodes (*p* < 0.05) (Figure 2B). The metastasized lymph nodes stained with HE showed that no obvious morphological differences were noted in metastasized lymph nodes of the F_ANXA7-control,_ F_ANXA7-down_, and P_ANXA7-control_ groups (Figure 2C).

### Expression and subcellular localizations of Annexin A7 protein in mouse xenografts

**Figure 3:**
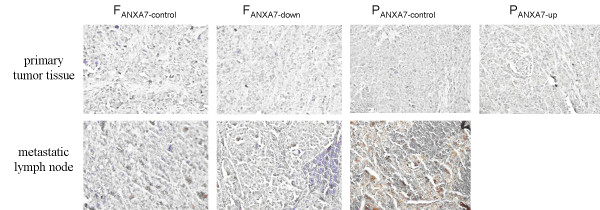
**Immunohistochemistry analysis of Annexin A7 expression in primary tumor and metastatic lymph node tissues.** The subcellular localization of Annexin A7 protein in primary tumor cells was mainly in the cytosol and partially in the cell membrane, while Annexin A7 expression in lymph node metastatic tumors was only localized in the cytosol. All magnifications are × 400.

Next, we analyzed the expression and subcellular localization of Annexin A7 in mouse primary tumor tissue and metastatic lymph nodes using immunohistochemistry. We found that the subcellular localization of Annexin A7 protein in primary and lymph node-metastasized tumors was mainly in the cytosol, and some of the Annexin A7 protein was partially localized in the nuclei and cell membrane (Figure 3). The expression intensity of Annexin A7 protein between high and low lymph node-metastasized tumors was also different: there was lower Annexin A7 expression in primary F_ANXA7-down_ tumor cells than in F_ANXA7-control_ tumors (*p* < 0.05). In addition, Annexin A7 expression was greater in primary tumors of P_ANXA7-up_ cells than in those of P_ANXA7-control_ cells (*p* < 0.05). Furthermore, Annexin A7 expression was greater in lymph node-metastasized tumors derived from F_ANXA7-down_ cells than from F_ANXA7-control_ cells (*p* < 0.05). Likewise, Annexin A7 expression was greater in F_ANXA7-control_ primary tumor cells than in lymph node-metastasized tumors (*p* < 0.05; Table 2).

**Table 2:** Annexin A7 protein expression in primary and lymph node metastasized tumors

**Tissue**	**N**	**Annexin A7 expression**				***p*-value 1**	***p*-value 2**
		**-**	**+**	**++**	**+++**		
F_ANXA7-control_ primary tumor	13	0	2	5	6	0.000^*^	0.001^◆^
lymph node metastasized tumor	16	10	1	4	1		
F_ANXA7-down_ primary tumor	34	8	22	3	1		0.000^■^
lymph node metastasized tumor	66	15	8	17	26	0.002^#^	
P_ANXA7-control_ primary tumor	11	5	5	1	0	0.000^Δ^	0.001^●^
Lymph node metastasized tumor	4	0	0	1	3		
P_ANXA7-up_ primary tumor	26	1	5	7	13		

*P*-value 1 shows the difference among different groups. *The expression of Annexin A7 in primary tumors of F_ANXA7-down_ cells vs. that of F_ANXA7-control_ cells. Δ The expression of Annexin A7 in primary tumors of P_ANXA7-up_ cells vs. that of P_ANXA7-control_ cells. # The expression of Annexin A7 in lymph node metastasized tumors of F_ANXA7-down_ cells vs. that of F_ANXA7-control_ cells.

*P*-value 2 shows the difference within the group. ^◆^The expression of Annexin A7 in primary tumors of F_ANXA7-control_ cells vs. that of lymph node metastasized tumors. ^■^The expression of Annexin A7 in lymph node metastasized tumors of F_ANXA7-down_ cells vs. that of primary tumors. ^●^The expression of Annexin A7 in lymph node metastasized tumors of P_ANXA7-control_ cells vs. that of primary tumors.

### Expression of Annexin A7 levels in high- and low-lymph node-metastasized primary tumors

**Figure 4:**
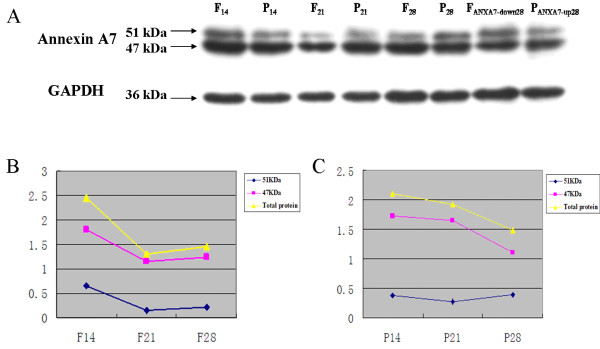
**Western blot analysis of Annexin A7 expression at different phases of tumor formation. (A)** Expression of Annexin A7 proteins (47 kDa and 51 kDa) at 14th, 21st, and 28th days after inoculation of Hca-F cells. **(B)** Quantification of Annexin A7 (47 kDa and 51 kDa isoforms) expression at 14th, 21st, and 28th days after Hca-F cell inoculation. **(C)** Quantification of Annexin A7 (47 kDa and 51 kDa isoforms) expression at 14th, 21st, and 28th days after Hca-P cell inoculation.

We investigated the time course of primary tumor formation in mice and found that on the 14th, 21st, and 28th days after tumor cell inoculation, both the 47 kDa and 51 kDa isoforms of Annexin A7 protein were detected in the “primary tumor” with different expression levels. Expression of the 47 kDa isoform in the F_ANXA7-down28_ group was less than that in the F_28_ group. The expression of the 51 kDa isoform in the P_ANXA7-up28_ group was greater than that in the P_28_ group (Figure 4A). In Hca-F cells with a high lymphatic metastasis potential, expression of the 47 kDa and 51 kDa isoforms was consistent with the total protein that was found on the 14th day, which was the highest point, and on the 21st day, which was the lowest level; while on the 28th day, the protein expression increased (Figure 4A, 4B). Whereas in Hca-P cells with a low lymphatic metastasis potential, the expression level of the 47 kDa isoform of Annexin A7 was consistent with the total protein, which decreased over time, while that of the 51 kDa isoform increased over time (Figure 4A, 4C).

## Discussion

In this study, we investigated the effects of Annexin A7 on HCC and lymphatic metastasis in a mouse model of lymph node metastasis by using the two mice hepatocarcinoma ascites syngeneic cell lines Hca-F and Hca-P with high and low lymphatic metastasis potential, respectively. The data showed that the lymph node metastasis rate was decreased from 36% to 0% after upregulation of Annexin A7 in Hca-P cells, but it increased from 77% to 100% after downregulation of Annexin A7 expression in Hca-F cells. Thus, the *in vivo* data implied that Annexin A7 may play an important role in HCC lymphatic metastasis and play a tumor suppressor function in HCC. A previous study has shown that Annexin A7 expression is lost in metastatic and local recurrent hormone-refractory prostate cancer compared to primary tumors [10]. Srivastava *et al.* reported the knockout of the Annexin A7 gene in mice to investigate the involvement of Annexin A7 in Ca^2+^ signaling in secreting pancreatic β cells and its function in the control of cancer development [11,12]. Annexin A7 has been shown to be a tumor suppressor in hormone-relevant prostate and breast cancers [10-15]. In prostate cancer, Annexin A7 as a tumor suppressor could be through inhibition of pathologic androgen signaling and dysfunctional retinoblastoma 1, PTEN, and p53 activity. Annexin A7 could also be associated with its mediation of exocytosis and secretion in prostate cells and possibly in other cancers [14]. In addition, haplo-insufficiency of Annexin A7 expression appears to drive disease progression to cancer because the genomic instability could lead to a discrete signaling pathway to reduce expression of the other tumor suppressor genes, DNA-repair genes, or apoptosis-related genes [12]. Some work regarding Annexin A7 from our laboratory clearly showed that the Annexin A7 gene is associated with lymph node metastasis and progression of HCC [5-7,16-19]. However, the tumor suppressor mechanisms of Annexin A7 in HCC have not yet been elucidated. Future studies will investigate Annexin A7 expression *ex vivo* before Annexin A7 expression is used to control HCC progression in the clinic.

Immunohistochemistry experiments showed that the subcellular localization of Annexin A7 protein in both the primary and lymph node-metastasized tumors was mainly in the cytosol, with some in the nuclei and cell membrane; while the level of Annexin A7 expression in the tumors was associated with their metastasis potential. Our current study demonstrated that the subcellular localization of Annexin A7 protein may be involved with lymph node metastasis of HCC. Meanwhile, Asma *et al.* found that Annexin A7 protein can be localized in the cytosol, on the cell membrane, or on the cytoskeleton [17]. Furthermore, Rick *et al.* detected both of the Annexin A7 isoforms (47 kDa and 51 kDa) in a diabetes-related animal model. Diabetic wild-type animals showed reduced levels of the 47 kDa protein isoform. During brain development, Annexin A7 expression changes from the cytoplasm to the nuclei, and the subcellular distribution of Annexin A7 protein depends on the cell type in the adult central nervous system [20]. In this study, we found that Annexin A7 expression was different in metastasized lymph nodes and primeval tumor cells derived from Hca-P and Hca-F cells. This disparity illustrates that the Annexin A7 gene plays an important role in high and low lymph node metastasis. This result was supported by a study that disclosed that the loss of Annexin A7 is an important factor in distant metastasis of gastric cancer [21]. In addition, altered expression of Annexin A7 could affect the tumor stage and survival in hormone-refractory human prostate and breast cancers [22-24]. Molecularly, Annexin A7 can regulate cellular exocytosis [25,26], and the latter event was associated with tumorigenesis [27]. Annexin A7 can also modulate neoangiogenesis and tumor invasiveness through its involvement in VEGFR1 signaling [28]. Ras proteins control at least three crucial signaling networks, including anchorage independence, survival, and proliferation protein dysregulated pathways, such as Annexin A7 [29]. Annexin A7 can translocate from the cytoplasm to the cellular membrane in cultured cells after damage, apoptosis, and treatment with Ca^2+^-ionophore [30]. The 47 kDa isoform of Annexin A7 is expressed in astrocyte-derived C6 rat glioblastoma cells, which is in contrast to human brain tissues [31]. Both isoforms appear in red blood cells, heart muscle, and the brain [31-35]; different isoforms with a tissue-specific distribution may indicate different functions of Annexin A7 [34]. Our experiments showed that both the 47 kDa and 51 kDa isoforms of Annexin A7 occurred in hepatocarcinoma tissues. In Hca-F cells with a high metastasis potential, the 47 kDa isoform was abundant; whereas in Hca-P cells with a low metastasis potential, the 51 kDa isoform was dominant. In addition, the expression of the 47 kDa and 51 kDa isoforms varied over time; thus, these data suggest that both isoforms play different roles in HCC progression. Afterwards, we detected Annexin A7 expression in mouse xenografts from primary and secondary tumors and found that the expression levels of Annexin A7 in tumors were reversely associated with their metastasis potential, indicating that Annexin A7 does play a role in suppression of tumor metastasis *in vivo*. These data demonstrate that Annexin A7 functions as a tumor suppressor gene in hepatocarcinoma and could be further evaluated as a novel therapeutic target for hepatocarcinoma.

In summary, our current data demonstrate that the dysregulation of Annexin A7 is an important factor associated with lymph node metastasis of HCC. Further mechanistic studies will provide more insight into Annexin A7 tumor suppressor function for potential diagnostic and therapeutic uses.

## Conclusion

In summary, our study indicated that Annexin A7 expression was able to inhibit HCC lymph node metastasis, indicating that the Annexin A7 gene might play an important role in the process of tumor lymph node metastases.

## Competing interests

No competing financial or personal interest in any company or organization is reported.

## Authors’ contributions

JY participated in the design of the study and carried out the molecular genetics studies, gene transfection experiment, western blot analysis and animal experiments; and drafted the manuscript. CW carried out immunohistochemistry analysis and animal experiments. WS and WB participated in the cell culture and animal experiments. ZJ and GH participated in the sequence alignment, RT-PCR assay and performed the statistical analysis. TJ conceived the study, participated in its design and coordination, and helped to draft the manuscript. All authors read and approved the final manuscript.

## Pre-publication history

The pre-publication history for this paper can be accessed here:

http://www.biomedcentral.com/1471-2407/13/522/prepub
